# Dynamic brain activity states of memory impairment in stroke patients with varying motor outcomes

**DOI:** 10.3389/fnagi.2023.1294009

**Published:** 2023-11-17

**Authors:** Caihong Wang, Jingchun Liu, Jun Guo, Shaoqiang Han, Peifang Miao, Ying Wei, Yingying Wang, Xin Wang, Zhen Li, Kangkang Xue, Kaiyu Wang, Jingliang Cheng

**Affiliations:** ^1^Department of MRI, The First Affiliated Hospital of Zhengzhou University, Zhengzhou, Henan, China; ^2^Engineering Technology Research Center for Detection and Application of Brain Function of Henan Province, Zhengzhou, Henan, China; ^3^Engineering Research Center of Medical Imaging Intelligent Diagnosis and Treatment of Henan Province, Zhengzhou, Henan, China; ^4^Department of Radiology, Tianjin Key Laboratory of Functional Imaging, Tianjin Medical University General Hospital, Tianjin, China; ^5^Department of Radiology, Tianjin Huanhu Hospital, Tianjin, China; ^6^Department of Interventional Radiology, The First Affiliated Hospital of Zhengzhou University, Zhengzhou, China; ^7^MR Research China, GE Healthcare, Beijing, China

**Keywords:** cognitive, dALFF, fMRI, intrinsic brain activity, sALFF, stroke, temporal dynamic

## Abstract

**Introduction:**

The objective of this study was to characterize the alteration patterns of dynamic spatiotemporal activity in chronic subcortical stroke patients with varying motor outcomes, while investigating the imaging indicators relevant to the assessment of potential cognitive deficits in these patients.

**Methods:**

A total of 136 patients and 88 normal controls were included in the analysis of static and dynamic intrinsic brain activity, determined by amplitude of low-frequency fluctuations.

**Results:**

The findings unveiled that subcortical stroke patients exhibited significantly aberrant temporal dynamics of intrinsic brain activity, involving regions within multiple brain networks. These spatiotemporal patterns were found to be contingent upon the side of the lesion. In addition, these aberrant metrics demonstrated potential in discerning cognitive deficits in stroke patients with memory impairment, with the dynamic indices exerting more influence than the static ones. The observe findings may indicate that subcortical stroke can trigger imbalances in the segregation and integration of spatiotemporal patterns across the entire brain with multi-domain networks, especially in patients with poor motor outcomes.

**Conclusion:**

It suggests that the temporal dynamics indices of intrinsic brain activity could serve as potential imaging indicators for assessing cognitive impairment in patients with chronic subcortical stroke, which may be associated with the motor outcomes.

## Introduction

Motor and cognitive impairments are prevalent after stroke (Zhao et al., 2018; Shekhar et al., 2021; Chen et al., 2022), even in patients with mild strokes (Einstad et al., 2021). Compared to motor function injuries, cognitive function injuries have the characteristics of being concealed and delayed, resulting in patients with cognitive impairment not seeking medical treatment promptly. Especially the occurrence of mild cognitive impairment in chronic stage is frequently ignored by patients families, and may gradually develop into irreversible severe cognitive decline. Additionally, patients with motor function impairments may also suffer from concurrent cognitive impairments, specifically memory and executive function (Van Der Werf et al., 2003; Delavaran et al., 2017). This may be attributed to the fact that the brain is a complex network consisting of multiple subnetworks serving different functions (Wang et al., 2014). A structural lesion may disrupt the optimal balance between integration and segregation in the entire brain, thereby significantly contributing to the widespread abnormalities in network coherence associated with behavioral deficits.

Memory is considered a fundamental in human cognitive activities. It plays a crucial role in various cognitive processes, including learning, problem-solving, reasoning, intellectual performance, other significant aspects of human cognition (Mecklinger, 2010). Memory allows us to learn from past events, make decisions, and navigate through our daily lives (Mecklinger, 2010). The absence of memory would significantly hinder our capacity to acquire new knowledge, identify individuals, retrieve crucial information, or even maintain self-awareness. The previous studies have revealed that the decline in memory has a significant impact on the long-term cognitive functions in patients after a stroke (Van Der Werf et al., 2003; Al-Qazzaz et al., 2014; Gorelick and Nyenhuis, 2015; de Lima Ferreira et al., 2019). In this study, we mainly explored the characteristics of memory impairment and the change patterns of brain activity in stroke patients with different motor outcomes for identification and prevention of concurrent impair with behavior decline.

Research has demonstrated the significance of regional spontaneous neural characteristics in comprehending neuropathological and neurophysiological conditions (Tsai et al., 2014). The analysis of amplitude of low-frequency fluctuations (ALFF) is a reliable and reproducible resting-state functional magnetic resonance imaging (rs-fMRI) technique utilized for measurement of regional spontaneous neuronal activity (Zang et al., 2007). The ALFF has been widely used to locate abnormal functional activities associated with various brain disorders by calculating the amplitude of low-frequency oscillation of each voxel (Zang et al., 2007; Cao et al., 2018; Li et al., 2019; Cui et al., 2020; Wu et al., 2023). Prior study have illustrated that local properties (i.e., ALFF) play a crucial role in comprehending the neural pathology associated with stroke (Quan et al., 2022). The identification of localized activity through ALFF in a distinct cortical region may hold particular significance in reflecting specific aspects of functional processes within the network (Rubinov and Sporns, 2010; Wang P. et al., 2019). However, the traditional rs-fMRI technique studies are usually focused on static metrics (e.g., static ALFF, sALFF), which may overlook the valuable information within the temporal features of neural fluctuations. The activity of the brain is inherently dynamic, which is reflected in its temporal variability. Dynamic ALFF (dALFF) is derived from time-variant intrinsic brain activity, effectively highlights aberrant functional activity in psychological and cognitive disorders. The dALFF metric takes into account not only the average amplitude of these low-frequency fluctuations but also their temporal variability. By examining the dynamic changes in the ALFF, we can gain insights into the dynamic characteristics of brain activity and its relationship with various cognitive processes and neurological disorders. Previous studies have demonstrated that patients with stroke exhibit dynamic alterations in spontaneous neural activity across multiple brain networks (Guo et al., 2019; Ma et al., 2021; Wang et al., 2022), indicating the potential utility of dynamic local metrics as predictive tools for functional outcomes in stroke patients. This dynamic attribute emphasizes the materiality of an intact neuroanatomical connectome in facilitating effective information exchange among interconnected regions.

Additionally, the impact of stroke on cognition was closely associated with the lesion-side (Biesbroek et al., 2017; Zhao et al., 2018). It was found that there is heterogeneity in both brain structure and function of stroke patients with different lesion-sides (Diao et al., 2017; Wang C. et al., 2019). This may be attributed to the fact that despite exhibiting nearly identical structural characteristics in the left and right hemispheres, they exhibit significant functional disparities. Specifically, the localization of specific behavioral functions in one hemisphere is a salient feature of the human brain known as its dominant hemisphere. The left hemisphere is typically dominant in the majority of adults due to right-handedness. Consequently, variations in stroke-side of the dominant or non-dominant hemispheres may give rise to diverse characteristics and degrees of impairment in behavioral functionality, accompanied by heterogeneous damage and reorganization processes affecting both brain structure and function. However, it still lacks a comprehensive understanding of the spatiotemporal patterns in intrinsic brain activity related to memory impairment among patients with varying motor outcomes on different lesion sides.

In this study, we utilize static and dynamic metrics (i.e., sALFF, dALFF) to characterize the spatiotemporal patterns of alteration in chronic subcortical stroke patients with different motor outcomes. In addition, to investigate the characterize of significant heterogeneity in memory indicators and internal brain activity patterns in patients with different lesion-sides compared to healthy control group. The relationship between these indicators and memory impairment was also investigated, identifying reliable imaging markers for evaluation of potential memory dysfunction in these patients with diverse motor outcomes.

## Materials and methods

### Participants

The experimental protocol received approval from the medical research ethics committees of the First Affiliated Hospital of Zhengzhou University, Tianjin Medical University General Hospital, and Tianjin Huanhu Hospital. Additionally, it received approval and pre-registration from the Chinese Clinical Trial Registry (ChiCTR1900027064). Every participant provided written informed consent.

The inclusion criteria for stroke patients were as follows: (a) first-onset ischemic stroke with motor deficits in both upper and lower extremities at the onset of stroke; (b) a single lesion involving the motor pathway, such as at the level of the internal capsule or pontine, in stroke patients; and (c) right-handedness prior to stroke onset. Exclusion criteria included: (a) recurrent stroke based on clinical history and MRI evaluation; (b) any other brain abnormalities detected on MR images; (c) white matter hyperintensities greater than 1 were assessed using the modified Fazekas score (Fazekas et al., 1987); and (d) a history of any other neurological or psychiatric disorders.

**Figure 1:**
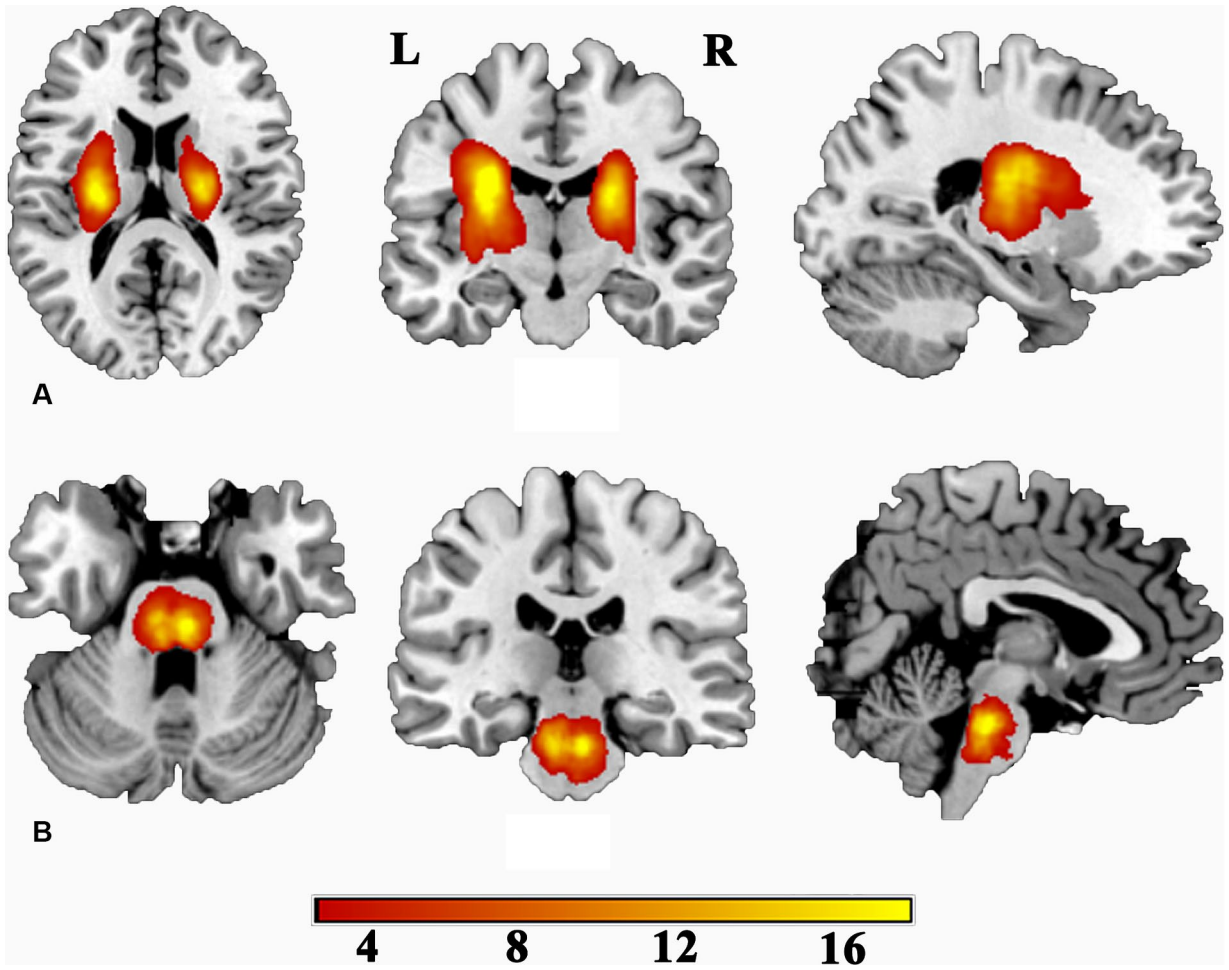
Lesion incidence map for patients with subcortical stroke. **(A)** For patients with stroke at the level of the internal capsule. **(B)** For patients with stroke at the level of the pontine. L, left; R, right. Color indicates the lesion incidence frequency.

The sample size estimation was conducted using G* Power software (version 3.1). Parameters were set as follows: two-tailed statistical test for the difference between two independent means, effect size *d* = 0.8, alpha error probability = 0.05, power = 0.95, and allocation ratio = 1. The calculated sample size of each group was 42, and a total of 136 eligible patients consented to participate in this study. Among them, there were 71 patients with left-hemisphere stroke (50 at the level of the internal capsule and 21 at the level of the pontine) and 65 patients with right-hemisphere stroke (46 at the level of the internal capsule and 19 at the level of the pontine). No randomization was performed to allocate subjects and no blinding were performed, as only two groups of subjects (patient and normal control) was recruited in this study. Additionally, the enrolled subjects were chronic ischemic stroke and did not undergo specific treatment protocols. 88 individuals without any medical history were recruited as normal controls (NC). A lesion incidence map of stroke patients is presented in Figure 1. In detail, the localization of the stroke was determined by two experienced neuroradiologists using three-dimensional T1-weighted images (3D-T_1_WI). Initially, we standardized the 3D-T_1_WI to the Montreal Neurological Institute (MNI) space as a reference. Subsequently, we manually delineated the lesions slice by slice on the normalized 3D-T_1_WI utilize MRIcron software.^1^ This process resulted in generating a lesion mask for each participant. Finally, the lesion masks of stroke patients were superimposed onto the MNI template to create a lesion incidence map of stroke patients.

### MRI data acquisition

MRI data were acquired from participants using 3.0-Tesla MR scanners at three hospitals, namely The First Affiliated Hospital of Zhengzhou University and Tianjin Medical University General Hospital (both equipped with a Discovery MR750 scanners from General Electric, Milwaukee, WI), and Tianjin Huanhu Hospital (equipped with a Magnetom Trio Tim MR scanner from Siemens, Erlangen, Germany). All participants were instructed to keep their eyes closed and remain still during the scanning procedure. Multi-parametric MRI including diffusion-weighted images (DWI), T_1_- and T_2_-weighted image (T_1_WI and T_2_WI), and T_2_ fluid-attenuated inversion recovery (T_2_-FLAIR) images were acquired to facilitate identification of the stroke lesion, recurrent stroke, white matter hyperintensity, and other brain abnormalities.

The images of subjects acquired from both Discovery MR750 scanners were obtained using identical parameters. For the MR750 scanners, rs-fMRI data were acquired using a gradient echo single-shot echo-planar imaging sequence with the following parameters: time of repetition (TR)/time of echo (TE) = 2,000/41 ms; field of view (FOV) = 220 mm × 220 mm; matrix size = 64 × 64; flip angle = 90°; slice thickness = 4 mm with a gap of 0.5 mm between slices; total number of slices = 32; and time points acquired per subject was set to be 180. Sagittal 3D-T_1_WI were utilized to acquire anatomical images, with TR/TE = 8.2/3.2 ms; FOV = 256 mm × 256 mm; matrix = 256 × 256; slice thickness = 1.0 mm, no gap; and a total of 188 slices resulting in isotropic voxel measuring at a resolution of 1-mm^3^. For the Trion Tim scanner, rs-fMRI data were acquired using an echo-planar imaging sequence with TR/TE = 2,000/30 ms, flip angle = 90°, FOV = 220 mm × 100 mm, matrix size = 64 × 64, thickness = 3 mm, gap = 1 mm, slices = 36, and volumes = 180. The 3D-T_1_WI was acquired using the magnetization prepared rapid acquisition gradient echo (MPRAGE) sequence with the following imaging parameters: TR/TE = 2,000/2.26 ms, flip angle = 9°, FOV = 256 mm × 232 mm, matrix = 256 × 232, thickness = 1 mm, and slices = 192, resulting in a 1-mm^3^ isotropic voxel.

### Neurological assessments

The National Institutes of Health Stroke Scale (NIHSS) was employed for the evaluation of global neurological deficits. Fugl-Meyer assessment of the whole extremity (WE_FM) was conducted to assess motor deficits in the chronic subcortical stroke patients, encompassing both upper- and lower-extremity motor function evaluation. According to the WE_FM scores, the patients were categorized into two subgroups: partial recovery (PR, WE_FM < 100) and complete recovery (CR, WE_FM = 100) (Zhang et al., 2014; Liu et al., 2023). The Rey Auditory Verbal Learning Test (RAVLT) was used to evaluate memory function (Ferreira Correia and Campagna Osorio, 2014), encompassing both verbal short-term memory (VSTM) and verbal long-term memory (VLTM).

### fMRI data processing

All pre-processing was performed using the Data Processing & Analysis of Brain Imaging (DPABI) toolbox (Yan et al., 2016),^2^ with the following steps: (1) the first 10 time points from each subjects were discarded to ensure steady-state magnetization; (2) the remaining time points were corrected to consistent image timing across slices; (3) a realignment procedure for head-motion correction was performed to exclude a part of participant data exhibiting head motion >2.0 mm or translation >2.0° rotation. Additionally, scrubbing was implemented with a frame-wise displacement threshold of 0.5 in order to reduce motion-related artifacts; (4) the functional images were subsequently normalized to the standard Montreal Neurological Institute (MNI) templates using Diffeomorphic Anatomical Registration Through Exponentiated Lie algebra (DARTEL), followed by resampling to a voxel size of 3 mm × 3 mm × 3 mm; (5) detrending the data; (6) conducting linear regression analysis to regress out the effects of several covariates; and (7) applying temporal band-pass filtering within frequency range of 0.01–0.08 Hz.

### sALFF calculation

The sALFF was calculated using DPABI software in MATLAB. Specifically, the BOLD time series of a given voxel were first transformed into the frequency domain via a fast Fourier transform. The square root of each frequency in the power spectrum was then calculated and averaged across 0.01–0.08 Hz to obtain the sALFF value. Prior to statistical analyses, these values were *z*-transformed. All analyses were conducted at the whole-brain level.

### dALFF variance calculation

The ALFF was proposed to characterize the resting-state functional activity of each voxel in the brain. The time series was initially transformed into the frequency domain, and subsequently, the ALFF was computed by measuring power within the low-frequency range of 0.01–0.08 Hz. A sliding window approach was employed to compute the dALFF using the Dynamic Brain Connectome (DynamicBC) toolbox (Liao et al., 2014).^3^ A window size of 30 TRs (60 s) and a window overlap of 90% were selected. To ensure the robustness of our findings, we conducted repeated analyses of dALFF using additional window lengths of 22 TRs and 50 TRs. For each window, an ALFF map was computed and the variance across all windows was used to quantify dynamic dALFF. The resulting dALFF maps were transformed into *z*-scores for statistical analysis.

### Statistical analysis

The statistical analyses were performed using SPM 12^4^ and GraphPad Prism.^5^ Data normality was assessed using the Shapiro–Wilk statistic, while differences in age between patients and the NC group were detected using a two-sample *t*-test with Welch’s correction. The non-parametric Mann–Whitney test was employed to discern disparities in educational attainment between the patient and NC cohorts. Fisher’s exact test was used to examine sex differences between the groups. A one-sample *t*-test was conducted to assess intra-group patterns of sALFF and dALFF maps. A general linear model (GLM) was employed to compare the difference in sALFF and dALFF maps between stroke patients and NC group, with age, sex, years of education, and neuroimage scanning sites as covariates. The height level family wise error (FWE) method was utilized for multiple comparison correction (*p* < 0.05, cluster size >20 voxels). The non-parametric Kruskal–Wallis test with Dunn’s multiple comparisons was employed to compare the dALFF values among the PR, CR, and NC groups (*p* < 0.05). Additionally, the dALFF values from the areas exhibiting significant inter-differences in patients were extracted for the analysis based on a region of interest (ROI). The Cohen’s d statistic (Parker and Hagan-Burke, 2007) was employed to quantify the effect size (ES) of inter-group differences. One-way ANOVA with Tukey’s multiple comparisons test was utilized to detect differences in RAVLT scores among the PR, CR, and NC groups (*p* < 0.05).

Spearman correlation analyses were performed to explore the relationship between dALFF variability in these regions of inter-group differences and memory scale scores with a Benjamini-Hochberg-Yekutieli (BY) FDR method (Benjamini and Yekutieli, 2001) for multiple comparison correction (*p* < 0.05). Finally, receiver operating characteristic (ROC) curve analysis (Mandrekar, 2010; Chen et al., 2018) was performed to investigate the potential value of dALFF variability indicators as an imaging biomarker for distinguishing stroke patients from healthy individuals. The Youden’s index J (J = sensitivity + specificity – 1) was utilized to identify the optimal cut-off that balances sensitivity and specificity.

## Results

### Clinical characteristics

The demographic, clinical, and behavioral characteristics are presented in Table 1. There were no significant inter-group differences in age between left-stroke patients and normal controls (*p* = 0.7209) or between right-stroke patients and normal controls (*p* = 0.4213). There were significant inter-group differences in sex between left-stroke patients and normal controls (*p* = 0.0195), whereas no significant inter-group differences in sex were observed between the right-stroke group and normal controls (*p* = 0.4078). There were significant inter-group differences in the years of education between the left-stroke group and normal controls (*p* = 0.0005) as well as between the right-stroke group and normal controls (*p* = 0.0021).

**Table 1:** Demographic and clinical information of participants.

Variable	Patients with subcortical stroke		Normal controls (NC)	Stroke patients vs. NC (*p*-values)		PR vs. CR	
	Left stroke (*n* = 71)	Right stroke (*n* = 65)	*n* = 88	Left stroke vs. NC	Right stroke vs. NC	Left stroke	Right stroke
Age, y	56.0 ± 7.4 (43–75)	56.57 ± 7.7 (40–72)	55.6 ± 7.6 (40–75)	0.7209	0.4213	—	—
Sex (M/F)	53/18	41/24	49/39	0.0195^*^	0.4078	—	—
Education, y	9.5 ± 3.2 (5–15)	9.8 ± 3.7 (5–21)	11.3 ± 2.9 (5–15)	0.0005^*^	0.0021^*^	—	—
Lesion location	71 (50.7%)	65 (46.4%)	—				
NIHSS	1 (0–2)	1 (0–2)	—	—	—	—	—
WE_FM							
PR	82 ± 24.2 (19–99)	78 ± 21.7 (28–99)	—	—	—	—	—
CR	100 ± 0.0 (100)	100 ± 0.0 (100)	—	—	—	—	—
RAVLT							
VSTM			49.7 ± 8.4 (29–73)	—	—	—	—
PR	39.0 ± 10.4 (15–64)	39.0 ± 9.7 (18–56)	—	<0.0001^*^	<0.0001^*^	0.0991	0.0103^*^
CR	43.4 ± 8.4 (27–65)	46.5 ± 8.9 (34–73)	—	0.0008^*^	0.2111		
VLTM			11.6 ± 2.4 (5–15)				
PR	9.6 ± 2.9 (1–15)	8.7 ± 2.8 (2–14)	—	0.0010^*^	<0.0001^*^	0.4869	0.0002^*^
CR	10.4 ± 2.7 (4–15)	11.3 ± 2.3 (5–15)	—	0.0330^*^	0.8076		

CR, complete recovery; NIHSS, National Institutes of Health Stroke Scale; PR, partial recovery; RAVLT, Rey Auditory Verbal Learning Test; VLTM, verbal long-term memory; VSTM, verbal short-term memory; WE_FM, Fugl-Meyer Assessment of the whole extremity; Italics (*) indicate statistical significance.

**Figure 2:**
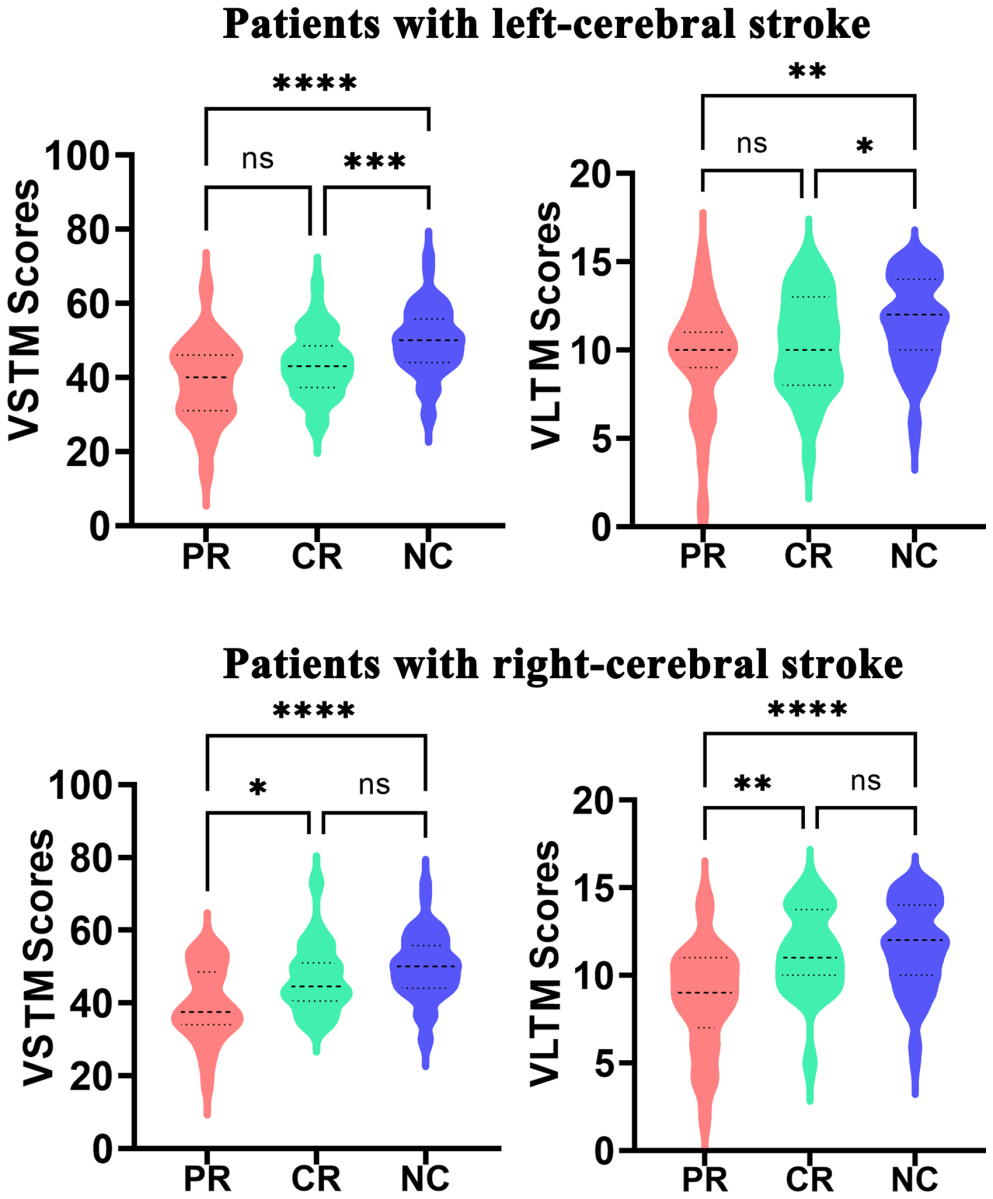
The significant inter-group differences in VLTM and VSTM among the PR, CR, and NC groups. CR, complete recovery; PR, partial recovery; NC, normal control; ns, none significant; VLTM, verbal long-term memory; VSTM, verbal short-term memory.

The RAVLT scores are displayed in Figure 2. The left-stroke patients with both PR (*P*_VSTM_ < 0.0001, *P*_VLTM_ = 0.0010) and CR (*P*_VSTM_ = 0.0008, *P*_VLTM_ = 0.0330) involvement exhibited poorer performance in the VSTM and VLTM tests compared to normal controls, while no significant difference was observed between the PR and CR groups (*P*_VSTM_ = 0.0991, *P*_VLTM_ = 0.4869). The right-stroke patients in the PR group exhibited significantly poorer performance on both the VSTM test (*P*_VSTM_ < 0.0001) and the VLTM test (*P*_VLTM_ < 0.0001) compared to normal controls, while there were no significant inter-group differences between the CR group and normal controls (*P*_VSTM_ = 0.2111, *P*_VLTM_ = 0.8076). Additionally, the right-stroke patients in the PR group performed significantly worse in both the VSTM test (*P*_VSTM_ = 0.0103) and the VLTM test (*P*_VLTM_ = 0.0002) compared to their counterparts in the CR group.

### sALFF changes

**Figure 3:**
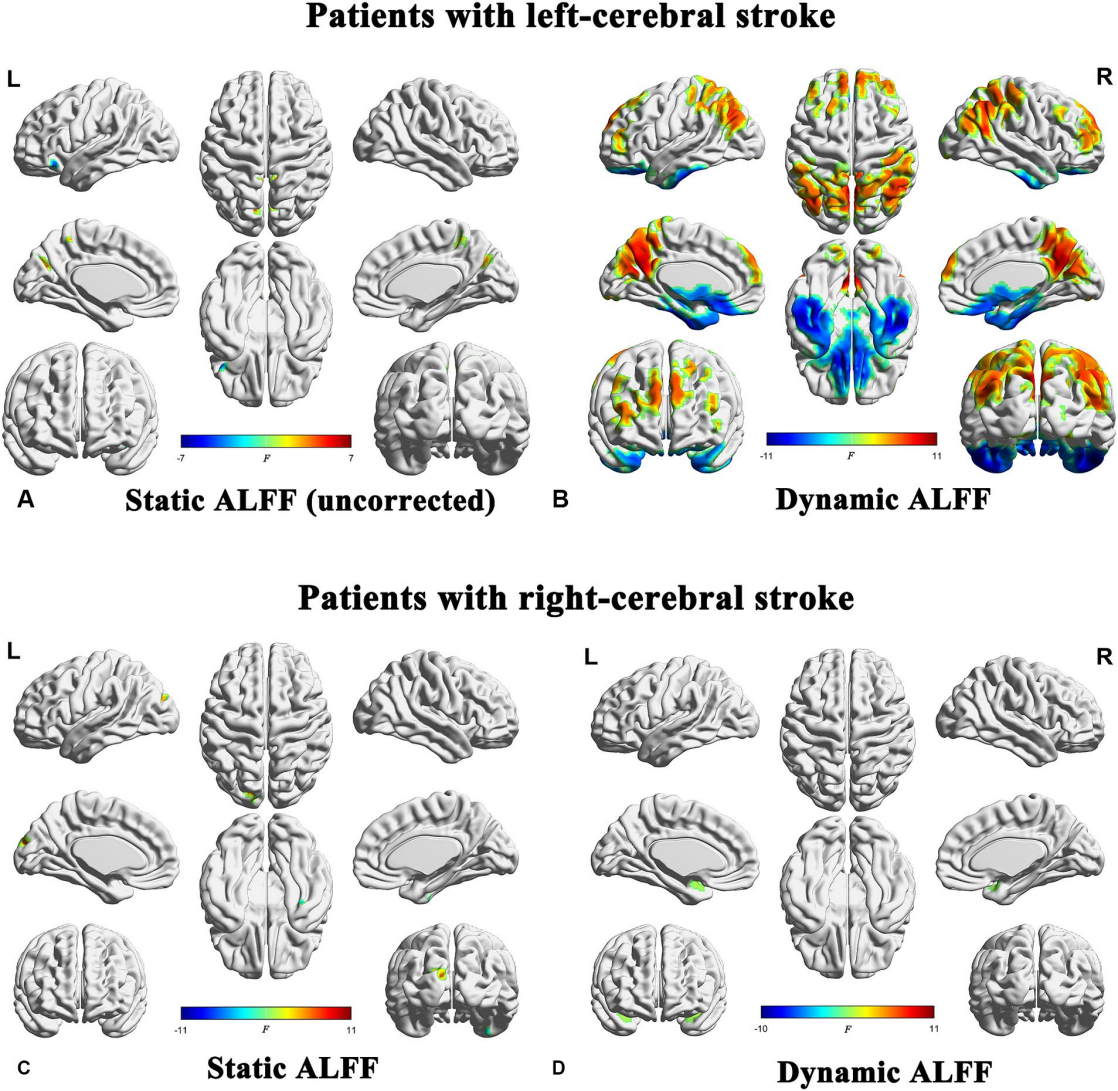
Brain regions showing significant differences in both sALFF and dALFF analyses between stroke patients and normal controls (FWE correction, *p* < 0.05). ALFF, amplitude of low-frequency fluctuations; L, left; R, right.

Significant differences in sALFF were observed between the right-stroke and NC groups, while no significant differences were detected between the left-stroke and NC groups after multiple comparison correction (FWE, *p* < 0.05, cluster size >50). In detail, there were no significant differences observed between the left subcortical stroke patients and the NC group after multiple comparison correction (FWE, *p* < 0.05). However, in order to observe the trend of ALFF in the left-stroke patients more accurately, we performed a comparison without multiple comparison correction (uncorrected, *p* < 0.001, cluster size >50). As a result, it was found that the left-stroke patients had increased ALFF in the bilateral cuneus and bilateral precuneus regions, while decreased ALFF was observed in left inferior orbitofrontal cortex (Figure 3A). Compared to the normal control group, chronic stroke patients with right-hemisphere lesions exhibited significantly increased sALFF in left superior occipital gyrus and significantly decreased sALFF in right fusiform (Figure 3C).

### dALFF changes

**Figure 4:**
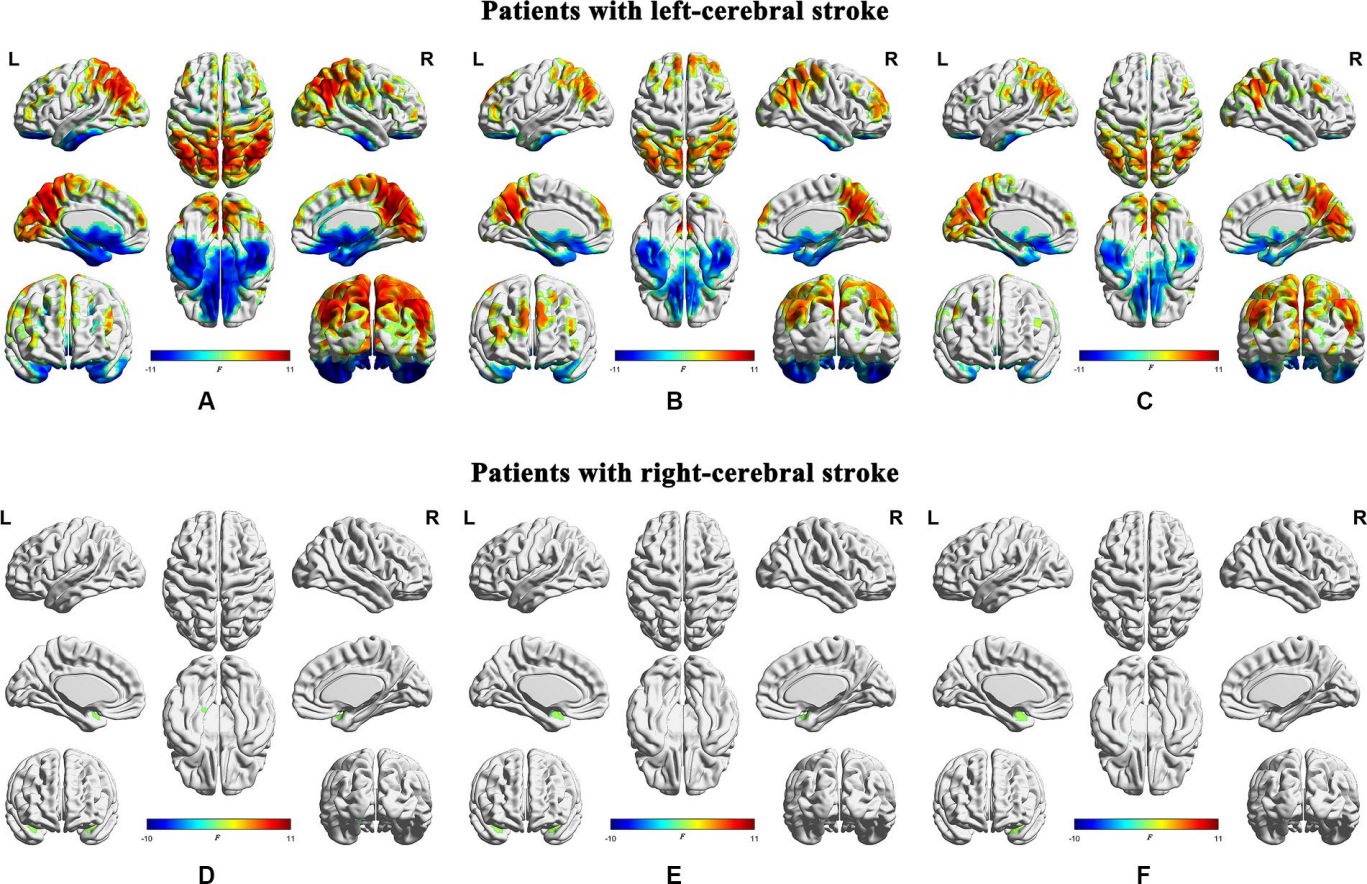
Validation results obtained with different window lengths. Compared with normal controls, patients with left-cerebral stroke showed significant dALFF variances, as illustrated in panels **(A)** (22 TRs), **(B)** (30 TRs), and **(C)** (50 TRs). By contrast, patients with right-cerebral stroke showed significant dALFF variances, as illustrated in panels **(D)** (22 TRs), **(E)** (30 TRs), and **(F)** (50 TRs) (FWE correction, *p* < 0.05). ALFF, amplitude of low-frequency fluctuations; L, left; R, right.

**Figure 5:**
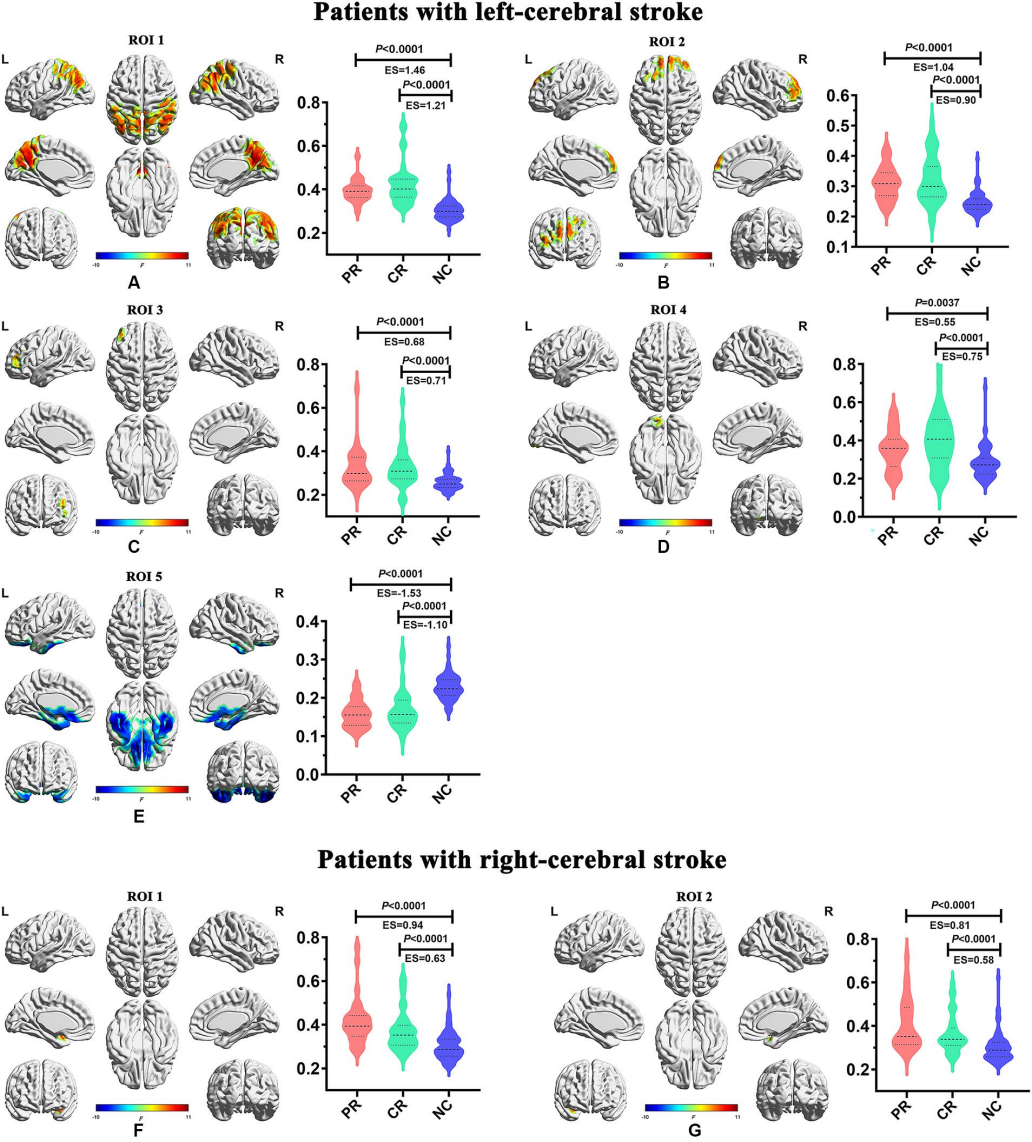
Inter-group differences of dynamic temporal variability among the PR, CR, and NC groups. CR, complete recovery; ES, effect size; L, left; NC, normal control; PR, partial recovery; R, right; ROI, region of interest.

The statistical significance of the differences in dALFF at a window length of 30 TRs between the stroke groups (both the left- and right-cerebral stroke) and the NC group was demonstrated in Figures 3B,D, 4B,E, while the significant inter-group differences among PR, CR, and NC were displayed in Figure 5 and Table 2. The validation results for both the left- and right-cerebral stroke, obtained using window lengths of 22 (Figures 4A,D) and 50 (Figures 4C,F) TRs respectively, are displayed in Figure 4.

**Table 2:** Brain regions with significant inter-group differences in dALFF among PR, CR, and NC groups.

Brain region	Cluster size (voxels)	Peak intensity	Peak MNI coordinates (x, y, z)
Left-cerebral stroke group			
ROI1	4,361	11.95	−6, −60, 21
ROI2	861	6.83	−9, 48, 42
ROI3	84	5.57	−39, 42, 21
ROI4	63	5.67	−15, −87, −12
ROI5	4,956	−16.72	−42, −27, −30
Right-cerebral stroke group			
ROI1	93	9.05	−36, 3, −18
ROI2	58	6.55	36, 6, −18

CR, complete recovery; dALFF, dynamic amplitude of low-frequency fluctuations; PR, partial recovery; ROI, region of interest.

In the main results (with a window length of 30 TRs), the left-stroke patients displayed significantly increased dALFF in comparison to the NC group, primarily in regions associated with dorsal attention network (DAN) and sensorimotor network (SMN) (ROI1, Figure 5A), involving bilateral precuneus, bilateral angular gyrus, bilateral inferior parietal gyrus, bilateral cuneus, bilateral superior parietal gyrus, right supramarginal gyrus, bilateral superior and middle occipital gyrus, bilateral middle temporal gyrus, right superior temporal gyrus, bilateral middle and posterior cingulum gyrus, bilateral postcentral gyrus, right precentral gyrus, and bilateral paracentral lobule; as well as frontal network (FN), involving bilateral superior and middle frontal gyrus (ROI2, Figure 5B), left middle and inferior frontal gyrus (ROI3, Figure 5C), and left lingual gyrus (ROI4, Figure 5D), part of the visual network (VN). These patients also exhibited decreased dALFF, primarily in the regions of frontotemporal network (FTN) and basal ganglia network (BGN) (ROI5, Figure 5E), encompassing bilateral superior, middle and inferior orbitofrontal cortex, bilateral rectus gyrus, bilateral inferior temporal gyrus, bilateral superior and middle temporal poles, bilateral fusiform gyrus, bilateral parahippocampus, bilateral hippocampus, bilateral pallidum, putamen and caudate.

In comparison with the NC group, the right-stroke patients showed significantly increased dALFF in regions of left temporal-insula network (TIN; ROI1, Figure 5F), encompassing the left superior temporal pole, superior temporal gyrus, and left insula; as well as right TIN (ROI2, Figure 5G), including the right superior temporal pole, superior temporal gyrus, and right insula.

### Correlation analysis

**Figure 6:**
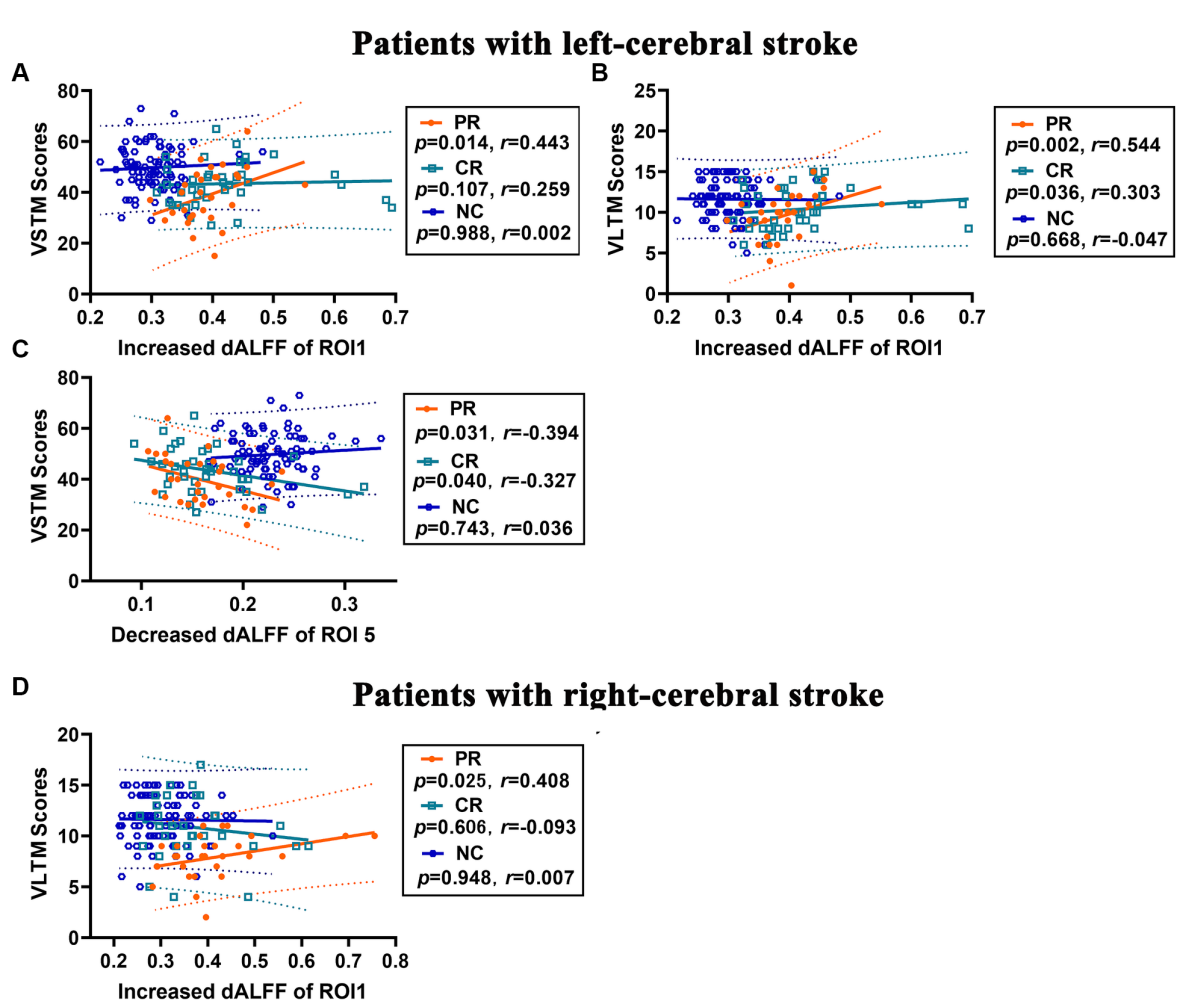
Correlation analysis between changes in dALFF and memory scores in stroke patients. **(A–C)** Correlations between increased dALFF of ROI1 and VSTM score **(A)** and VLTM score **(B)**, and between decreased dALFF of ROI5 and VSTM score **(C)** in patients with left-cerebral stroke. **(D)** Correlations between increased dALFF of ROI1 and VLTM score in patients with right-cerebral stroke. dALFF, dynamic amplitude of low-frequency fluctuations; ROI, region of interest; VLTM, verbal long-term memory; VSTM, verbal short-term memory.

In left-cerebral stroke patients, a significant positive correlation was observed between the increased dALFF of ROI1 and VSTM (Figure 6A) in PR group (*p* = 0.014, *r* = 0.443), yet no correlation was found in either the CR (*p* = 0.107, *r* = 0.259) or NC (*p* = 0.988, *r* = 0.002) group. As well as, the increased dALFF of ROI1 had a significant positive correlated with VLTM (Figure 6B) both in PR (*p* = 0.002, *r* = 0.544) and CR (*p* = 0.036, *r* = 0.303) group, while no significant correlation demonstrated in NC group (*p* = 0.668, *r* = −0.047). Additionally, the decreased dALFF of ROI5 displayed a significant negative correlation with VSTM (Figure 6C) both in PR (*p* = 0.031, *r* = −0.394) and CR (*p* = 0.040, *r* = −0.327) group, but no correlation was found in NC group (*p* = 0.743, *r* = 0.036).

In the right-cerebral stroke patients, a significant positive correlation was observed between the increased dALFF values of ROI1 and VLTM scores (Figure 6D) in PR group (*p* = 0.025, *r* = 0.408), while no significant correlation was found in CR (*p* = 0.606, *r* = −0.093) and NC (*p* = 0.948, *r* = 0.007) group.

### ROC curve analysis results

**Figure 7:**
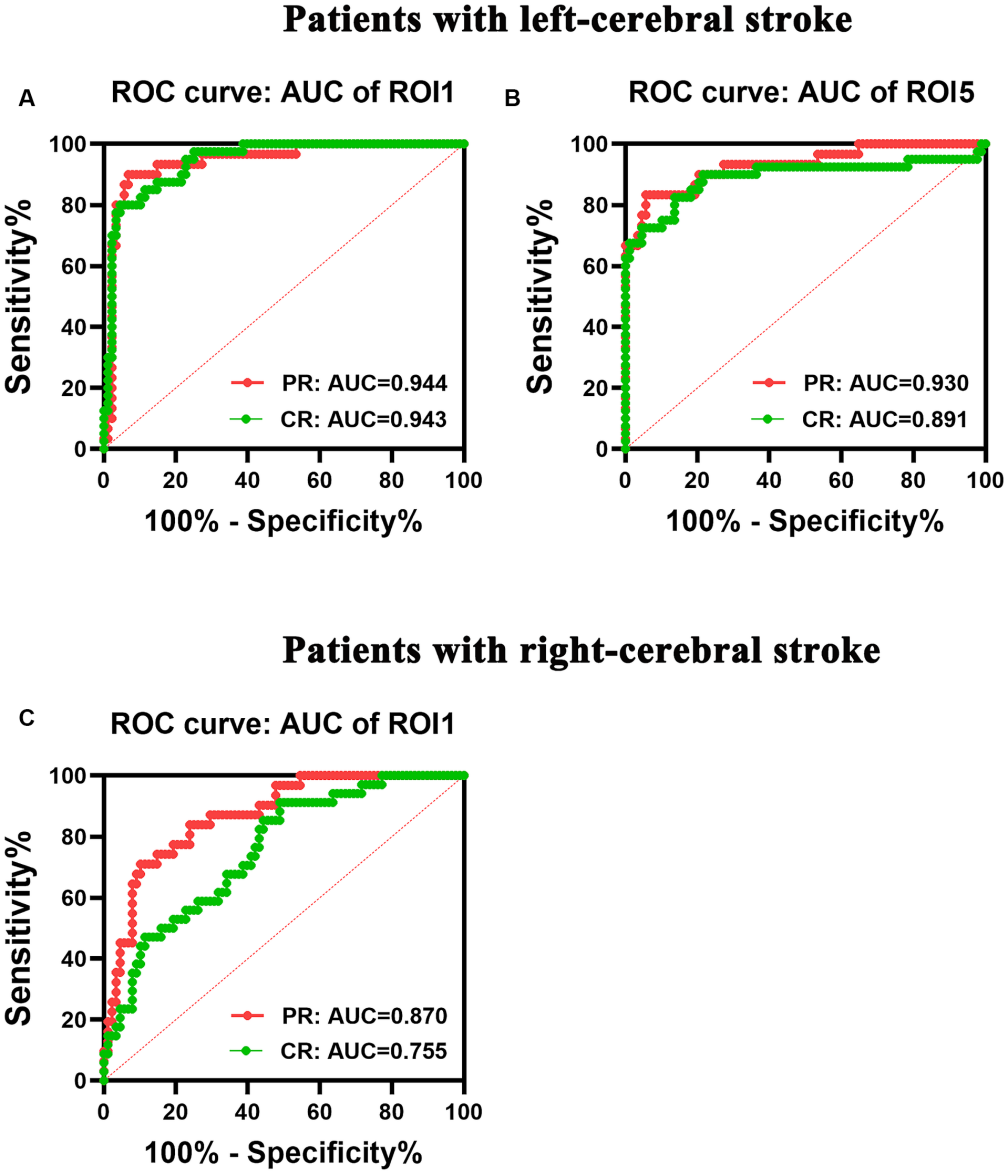
ROC analysis of the performance of changes in dALFF in patients with left- **(A,B)** and right-cerebral strokes **(C)**. AUC, area under curve; CR, complete recovery; dALFF, dynamic amplitude of low-frequency fluctuations; PR, partial recovery; ROC, receiver operating characteristic; ROI, region of interest.

The left-stroke group exhibited an area under curve (AUC) value of 0.944 and 0.943 in ROI1 with increased dALFF variability for PR and CR patients, respectively (Figure 7A), while the AUC values were 0.930 and 0.891 in ROI5 with decreased dALFF for PR and CR patients, respectively (Figure 7B). In the right-stroke group, increased dALFF of ROI1 in both PR and CR patients demonstrated AUC values of 0.870 and 0.755, respectively (Figure 7C).

## Discussion

In this study, we investigated the spatiotemporal patterns of intrinsic brain activity in subcortical stroke patients with different degrees of motor recovery, elucidating the correspondence between these metrics change and memory impairments. We observed that chronic subcortical stroke patients with motor pathways damage exhibited both short- and long-term memory impairments, particularly those who did not fully recover their motor function. This implies that the functional deficits induced by stroke in subcortical motor pathways affect not only the motor system but also the cognitive system. This is in line with the previous research indicating a reciprocal relationship between motor and cognitive impairments and repairs (Kannan et al., 2019; Einstad et al., 2021).

Our study revealed significant short-term and long-term memory impairments in both left- and right-cerebral stroke patients, with the memory impairment increasing as motor recovery worsened. Notably, particularly in left-cerebral subcortical stroke patients, substantial memory function impairment persists even in cases of good motor recovery. This finding aligns with a previous study conducted on mild strokes (Einstad et al., 2021) which revealed that around one-third of patients exhibited cognitive or motor deficits, along with concomitant impairment manifested in some survivors. Additionally, the author discovered a significant correlation between motor performance and deficits in memory. Generally, the capacity of short-term memory reflects the brain’s aptitude for rapidly assimilating and processing novel information and experiences, while long-term memory is associated with the ability to effectively encode and retrieve information over extended periods (Mecklinger, 2010). The memory functions are crucial for the daily functioning success of those stroke patients (Al-Qazzaz et al., 2014).

Compared to the normal controls, significant differences were observed in the characteristics of both static and dynamic spontaneous intrinsic brain activity throughout the entire brain in these patients who experienced partial recovery or complete recovery. It implies that chronic subcortical stroke patients, even those who have achieved good motor function recovery, still exhibit abnormal intrinsic brain activity at the global level. This may underlie their heightened vulnerability to cognitive dysfunction compared to age-matched controls. Additionally, this study found that the dynamic temporal variability exhibited greater sensitive than the static metrics in characterizing brain activity changes in patients with chronic subcortical stroke, particularly those with left-cerebral stroke. In detail, the static and temporal dynamic analyses showed similar alteration patterns of intrinsic brain activity in the right-stroke group. Nevertheless, no significant differences were observed in the static analysis in the left-stroke group when compared with the normal controls, whereas the patients displayed significant dynamic temporal variability involving the regions of multiple brain networks (FWE correction, *p* < 0.05). Notably, the tendency of change observed in the left-stroke patients was consistent with that revealed by the dynamic analysis when multiple comparison correction was not applied in the static analysis (uncorrected, *p* < 0.001). It suggest that temporal dynamic metrics are more effective in capturing uncontrolled yet recurring spontaneous intrinsic brain activity patterns than static indexes. It may be attributed to the static analysis assumes average quantities and does not account for the dynamic nature of brain activity in the resting state (Chang and Glover, 2010). However, the brain’s activity is inherently dynamic. This temporal dynamic variability is closely related to neural networks functionality and can reveal fluctuations in network interactions (Allen et al., 2014) and patterns of recurrent brain activity that are beyond the scope of static analyses (Liegeois et al., 2017; Qin et al., 2021). The findings of this study indicate that the dynamic neural properties metrics have greater potential than the static characteristics for diagnosing and predicting cerebral neurological function states in the stroke patients.

Notably, the dynamic temporal variability of neural circuits had a more significant impact in the left-stroke group than in the right-stroke group when compared to normal control group. This may be responsible for the greater memory dysfunction observed in patients with left subcortical strokes, even after adequate motor function recovery. Specifically, the left-stroke patients displayed significantly increased dynamic temporal variability of intrinsic brain activity across multiple brain networks, mainly in the regions of the dorsal attention network, sensorimotor network, frontal network, and visual network. In addition, they displayed significantly decreased dynamic temporal variability mainly in the regions of frontotemporal network and basal ganglia network. In contrast, the right-stroke patients only exhibited significantly increased dynamic temporal variability of intrinsic brain activity in the regions of bilateral temporal-insula networks. The activated intrinsic brain activity is associated with heightened neuronal excitability and metabolism, whereas reduced intrinsic brain activity signify inhibition of spontaneous neuronal activity. In the present study, the stroke patients displayed abnormalities in local temporal dynamic variability within the regions of primary perceptual networks (i.e., VN, SMN, BGN) and higher cognitive (i.e., FN, DAN, FPN, TIN) networks. These observations suggest that a subcortical stroke can trigger imbalances between the segregation and integration of spatiotemporal patterns of neural activity within the whole brain network. This finding is consistent with prior neuroimaging studies based on function and structure connection, which demonstrated that dysfunction induced by the subcortical stroke involves multiple-domain functional networks (Wang et al., 2014, 2021, 2022; Wang C. et al., 2019). The regions of advanced cognitive control network (i.e., FN) are responsible for externally directed higher-order cognitive function and diverse demanding tasks, such as guiding fine motor function (Liang et al., 2016; Wen et al., 2018). This may support the existence of a reciprocal relationship between motor and cognitive impairments and their respective repair mechanisms.

This study further revealed a significant correlation between the altered dynamic temporal variability and short- and long-term memory scores in both left- and right-stroke patients with varying degrees of motor recovery. This implies that the temporal dynamic variances indicators hold the potential to detect memory dysfunctions in chronic subcortical stroke patients, especially in the group of patients with poor motor outcomes. Additionally, the ROC curve analysis found that the altered regions of dALFF, which had exhibited significant correlations with memory, demonstrated notable AUC values in both left- and right-stroke patients, particularly those with partial recovery of motor function. It may suggest that the temporal dynamic variance indicator has significant potential in distinguishing chronic stroke patients with memory deficits from normal controls.

Additionally, our findings indicate significant heterogeneity in the left- and right- hemispheres stroke when compared to normal controls, both in terms of temporal dynamic variability and memory function. The precise neural mechanisms of the lesion-side effect on these indicators following a subcortical stroke remain unclear; yet, there may be several potential explanations. The right-handed subcortical stroke patients with lesions in the left hemisphere showed more extensive temporal dynamic variances than those in right-sided patients when compared with normal controls. These findings suggest that left-sided stroke patients have a greater imbalance of spatiotemporal patterns of intrinsic brain activity in the whole brain than right-sided patients, which is partly consistent with previous studies reporting more extensive changes in volumetric structural covariance and functional networks in the left-sided patients than in those with right-sided strokes (Wang C. et al., 2019; Wang et al., 2022). This may be attributed to the fact that the right-handedness stroke patients with lesions in left-dominant hemisphere demonstrate lower proficiency in performing daily tasks and exhibit more severe dysfunction than lesions in the right-non-dominant. Moreover, it is generally observed that the extent of brain injury correlates with the level of brain reorganization. In the study, left-stroke patients with good outcomes still exhibited considerable abnormalities in both their long-term and short-term memories, whereas right-stroke patients with equivalent motor recovery did not exhibit any notable deviations from normal controls in terms of memory function. Hence, a potential neural mechanism that can explain the heterogeneity of intrinsic brain activity resulting from the lesion-side may be the difference in severity of brain damage between the dominant and non-dominant hemispheres. Another potential explanation could be associated with the functional asymmetry between the left and right-cerebral hemispheres (Chiu and Damasio, 1980; Good et al., 2001; Corballis, 2014). The left hemisphere exerts a more significant role in executive functions for healthy right-handed individuals than the right hemisphere (Verstynen et al., 2005). Therefore, left-sided subcortical stroke patients with right-handedness may exhibit a higher potential for cortical reorganization, and more extensive disequilibrium in temporal dynamic variability than the right-sided strokes. Additionally, there existed a subtle disparity in the spatial distribution of lesions between lesion-side groups, which may also be associated with the impact of lesion-side on dynamic spatiotemporal patterns of intrinsic brain activity. These findings highlight the importance of considering the effect of the lesion-side and conducting individualized research on post-stroke brain dysfunction in future research endeavors. Certainly, it is essential to conduct more comprehensive research to explore the precise neural mechanisms of the lesion-side effect on the brain activity following subcortical strokes.

The present study is subject to certain limitations. Firstly, there was a wide variation in the time interval between stroke onset and MRI scan among participants, ranging from 6 to 70 months. This heterogeneity may have impacted the findings pertaining to specific post-stroke stages and could potentially limit the generalizability of our results. Future studies will focus on a specific post-stroke stage. Secondly, this study solely utilizes the ALFF as a metric to gauge the intrinsic brain activity. Future analyses will incorporate dynamic functional connection/network metrics with multiple indices to investigate the characteristics of behavioral dysfunctional in subcortical stroke patients. Thirdly, this was a cross-sectional study, and future research should consider conducting a longitudinal study to track the spatiotemporal patterns of intrinsic brain activity in these patients for early prediction. Last but not least, significant inter-group differences were observed in sex in left-stroke patients and the years of education in both left- and right-stroke patients compared to normal controls. Although we have adjusted for these factors in our analysis, they may still affect the specificity of our findings. Future studies will strive to augment the sample size to address potential confounding variables.

## Conclusion

The study revealed that chronic subcortical stroke patients with motor pathway damage exhibited both short- and long-term memory dysfunctions, particularly those with partial motor recovery. Additionally, these patients displayed atypical spatiotemporal patterns of intrinsic brain activity across the regions of multiple-domain brain networks, especially in terms of the dynamic temporal variability. The implication is that a subcortical stroke can disrupt the equilibrium between the segregation and integration of intrinsic neural activity within the entire brain network. Furthermore, this study indicated that the indicators of temporal dynamic variability could serve as valuable imaging biomarkers for evaluating potential cognitive dysfunction in chronic subcortical stroke patients with diverse motor outcomes.

## Data availability statement

The raw data supporting the conclusions of this article will be made available by the corresponding author, without undue reservation.

## Ethics statement

The studies involving humans were approved by the Institutional Review Boards of the First Affiliated Hospital of Zhengzhou University, Tianjin Medical University General Hospital, and Tianjin Huanhu Hospital. The studies were conducted in accordance with the local legislation and institutional requirements. The participants provided their written informed consent to participate in this study. Written informed consent was obtained from the individual(s) for the publication of any potentially identifiable images or data included in this article.

## Author contributions

CW: Conceptualization, Data curation, Formal analysis, Funding acquisition, Investigation, Methodology, Project administration, Resources, Supervision, Validation, Visualization, Writing – original draft, Writing – review & editing. JL: Data curation, Funding acquisition, Investigation, Writing – review & editing. JG: Conceptualization, Data curation, Writing – review & editing. SH: Methodology, Writing – review & editing. PM: Data curation, Investigation, Writing – review & editing. YWe: Data curation, Validation, Writing – review & editing. YWa: Methodology, Writing – review & editing. XW: Data curation, Investigation, Writing – review & editing. ZL: Project administration, Resources, Supervision, Writing – review & editing. KX: Formal analysis, Investigation, Writing – review & editing. KW: Formal analysis, Methodology, Writing – review & editing. JC: Funding acquisition, Project administration, Writing – review & editing.

## Figures and Tables

**Table:** 

3D-T1WI	3D T1-weighted images
AUC	Area under curve
BGN	Basal ganglia network
dALFF	Dynamic ALFF
DAN	Dorsal attention network
DARTEL	Diffeomorphic Anatomical Registration Through Exponentiated Lie
DPABI	Data Processing & Analysis of Brain Imaging
DWI	diffusion-weighted images
DynamicBC	Dynamic Brain Connectome
FOV	Field of view
FPN	Frontoparietal network
FWE	Family wise error
GLM	General linear model
LN	Language network
MNI	Montreal Neurological Institute
MPRAGE	Magnetization prepared rapid acquisition gradient echo
NC	Normal controls
NIHSS	National Institutes of Health Stroke Scale
pDMN	Posterior default mode network
PR	Partial recovery
RAVLT	Rey Auditory Verbal Learning Test
ROC	Receiver operating characteristic
ROI	Region of interest
sALFF	Static amplitude of low-frequency fluctuations
SMN	Sensorimotor network
T1WI	T1--weighted image
T2-FLAIR	T2 fluid-attenuated inversion recovery
T2WI	T2-weighted image
TE	Time of echo
TIN	Temporal-insula connection network
TR	Time of repetition
VLTM	Verbal long-term memory
VSTM	Verbal short-term memory
WE_FM	Whole extremity

## Footnotes

^1^ www.mricro.com

^2^ http://www.rfmri.org/dpabi

^3^v4.0, www.restfmri.net/forum/DynamicBC.

^4^ http://www.fil.ion.ucl.ac.uk/spm/

^5^Version 9.3.0, http://www.graphpad.com.
